# British Hospitals & National Insurance

**Published:** 1913-03-22

**Authors:** 


### BRITISH HOSPITALS & NATIONAL INSURANCE.

### Sir Henry Burdett Outlines a New Scheme of Co-operation.

### ANNUAL MEETING OF THE HOME HOSPITALS ASSOCIATION.

Speaking at the annual meeting of the Home Hospitals Association, reported below, Sir Henry Burdett said :

There never was a time in the history of hospitals in this country when it was more essential for the people of all classes to arouse themselves to a deep interest in the welfare of these voluntary institutions, because at the present time the condition of politics is such that even the meanest intelligence realises and regrets to know that party politics seem to so dominate politicians in these islands that in the House of Commons to-day they come before the country's needs. And the country's needs, under National Insurance, in regard to hospital accommodation, are becoming increasingly acute and severe every day. My subject then is British Hospitals and National Insurance. They have the closest inter-relation. You have seen all the trouble and the difficulties which have arisen with reference to the medical treatment of patients in their own homes. And it has been very remarkable that although we have had created some 15,000,000 of insured persons in these islands, no provision was made under the National Insurance Bill to give any one of them a day's treatment in a hospital. To say that is to show in a word the immense responsibility which was cast upon the voluntary hospitals by the passing of the National Insurance Act. Had it not been for the action which has been taken by the managers of the voluntary hospitals, the National Insurance scheme as passed must have entirely broken down, and it would have been impossible for it to have proceeded in the circumstances. The present position is that the voluntary hospitals are marking time. They have resolved to do their best, as I hope and believe everybody will do their best, now that we have it, to make National Insurance entirely successful. It was a great need of the nation, and when the necessary modifications have been introduced, and an amending Act or Acts have been passed, for a problem of this sort is so difficult that these must be brought in, matters will be better. If we take the German experience, we must realise that it may require many more than one amending Act. I believe that in Germany they have already had something like 32. The public spirit displayed by the hospital managers in deciding to deal with all applicants who come to them on the basis that they would only reject those who were suffering from ailments which could be properly treated by the panel doctors at their own homes, has left the voluntary hospitals with the responsibility of meeting all the in-patients' needs which may arise among insured persons.

#### The Position in London.

Now, in London there are two and a half millions of insured persons. They represent, according to the calculations of the actuaries, medical needs to the extent of 100,000 people a week, of whom 5,000 will require hospital in-patient treatment. Put another way, that means that the London voluntary hospitals which are effective for purposes of National Insurance contain some 10,000 beds. There are really about 11,000 beds in the hospitals of London, but the smaller institutions represent the 1,000 I have struck off, which may be ignored for the purposes of considering the in-patients who come as insured persons. When you bear in mind the proportion of our population in London to the whole number of the insured persons, and that the hospitals of London have to provide a great number of beds every year for severe cases that are sent up from the home counties and from various parts of the country, you will appreciate how great and serious is this claim, this relatively new claim, upon hospital beds in London, of 5,000 insured people every week. To make you realise it a little more clearly, I may point out that the average number of days' residence of in-patients is something like nineteen days. So that really it is quite possible that the demand for in-patient care on London hospital beds by insured persons may, when National Insurance settles down and comes into full working, not be less than 10,000 beds. I give the instance of London, but I could give the same thing to the whole country. I give it for London because it is an example which tersely and fully explains the position so far as the figures are concerned.

#### How the Hospitals have Saved the Act.

The hospital managers, not only in London, but in England and Wales and in Scotland, have courageously faced the position, and, recognising that their first duty is to the sick, have decided to use their utmost endeavours to meet the demands for in-patient treatment which insured persons must bring increasingly to the hospitals. It must be increasingly, because this army of insured persons is an organised army in the sense that every one of those persons has to be the patient of some medical man, and that if the hospitals are to take the severe cases, it means, in practice, to a large extent that the doctors become recruiting sergeants for the hospitals. And so the pressure on the beds is likely to be much greater than it has ever been before in the history of the voluntary hospitals in this country. It required great public spirit and great courage on the part of the hospital managers to undertake this responsibility as a tentative measure, extending over a period of a year, in order that every help might be given to the Government and to those responsible for carrying out the National Insurance scheme, with the view of diminishing the suffering and the confusion which must otherwise have arisen out of this large demand for this specially expensive kind of medical relief.

#### Reciprocity from the Government Called for.

You will see from what I have said that, financially, the action of the voluntary hospitals in this country has constituted them the backbone, financially, so far as medical benefit is concerned, of the National Insurance Act. It is based upon actuarial calculations, and every actuary will say that were it not for the voluntary hospitals National Insurance under the Act of 1911 must be financially a failure. Here, may I say in passing, that it is a very great disappointment to those who have the interests of the hospitals most nearly at heart to realise that there appears to be no gratitude for the courageous actions of the hospitals, if we may judge by the attitude of the Government and of Parliament. One great thing which would help us, and would immeasurably improve the position and make it much simpler to be administered, would be that steps should be taken to deal with the Poor-Law Commission's Report, a most important and pressing matter, which hampers and affects medical relief and many other social problems involved in it in every part of Great Britain. Then, again, the other day there was the case of legacy duty.

### The Truth about Legacy Duty.

The Chancellor of the Exchequer had a memorial from the British Hospitals Association, representing all the voluntary hospitals of Great Britain, asking him, having regard to the great financial pressure due to the work they were doing under the National Insurance Act, to help them not to lose their legacies by taking off the heavy legacy duty of 10 per cent. That legacy duty was imposed at a time when things were very different from what they are at the present day in this country. If that duty had to come up again before Parliament to be debated and dealt with it would never amount to 10 per cent., and I do not think it would ever be passed at all. What was the answer of the Chancellor of the Exchequer? Owing to the considerable loss of revenue, such was one reason he gave, he could not entertain the proposal. Now, I have been looking into this question of the loss of revenue, and I find, so far as the hospitals of the United Kingdom are concerned, it would represent only £80,000 a year, no more, for hospitals. And when I take all the great charities in the United Kingdom I find that the whole sum involved to the Exchequer is £150,000. I make that statement because I am perfectly certain that no Chancellor of the Exchequer or Members of Parliament representing the public, if dealing with this matter firmly, will ever continue to refuse to reconsider the whole question of legacy duty so far as the hospitals are concerned. There was one other reason given, and that was that very serious demands would follow concession. I have shown that the very serious demands which would follow concession, so far as charities were concerned, is only £70,000, making a total of £150,000. I have known most Chancellors of the Exchequer for a good many years, and it has been part of my privilege and work to be twenty years in the gallery on Budget night, and I make bold to say that we never had before a Chancellor of the Exchequer who, in the circumstances, would not take up this matter of the legacy duty and adjust it fairly and squarely on the merits and in the interests of all classes of the community, and in accordance with the wishes and the best interests of the people. I hope and believe that Mr. Lloyd George, when he really has all the facts before him, will follow some such course as that.

### Wanted, a Statesman.

The solution of the hospital problem really is not a difficult one for a statesman. We want a statesman. A statesman would realise, as at any rate Mr. John Burns realises, I am glad to say, that what this country needs is a system of co-operation which brings into direct relation and the closest intercourse for administrative purposes the whole of the agencies for the relief of the sick in London and throughout the country. I say that I am sure Mr. John Burns will entirely endorse this view, because his work as President of the Local Government Board in recent years fully confirms this. Mr. John Burns is one of the best administrators we have ever had at the Local Government Board, and he is a man of great practical common sense.

### Mr. John Burns at His Best.

In order to show the position in London, I may tell you what he has done in reference to two things: casuals and tuberculosis. Everybody for years has known of the very distressing scenes which have occurred on the Thames Embankment at night owing to casuals. Owing to the action of Mr. John Burns, taken so recently as April last, you may now go down on the Embankment at any hour of the night, and you will find it is clear of casuals altogether, and that the whole of the casuals of London are now provided for adequately and comfortably, and the old existing evils and abuses are entirely done away with. How has that been done? It has been done by powers vested in the Local Government Board under the Gathorne Hardy Act of 1867. It is [illegible]sible under that Act, by an Order of the Poor Law Board, which was issued under it, to combine into districts the unions and parishes of the Metropolis as the Local Government Board may think fit. Mr. John Burns issued an order in November 1911 forming a district coterminous with the existing Metropolitan Asylum district for the relief of the casual poor of the Metropolis. The order also provided that the Managers of the Metropolitan Asylums Board should be the managers of the new district. Prior to the issue of this Order each separate Metropolitan Board of Guardians was required to provide casual wards for destitute wayfarers, and there were twenty-eight separate casual wards in existence. Twenty-four of these casual wards were taken over by the Board on April 1, 1912, the remaining four being retained by the Boards of Guardians concerned and applied to other uses. The total accommodation of the twenty-four wards taken over is for 1,758 inmates.

### What Co-operation has Done Already.

During the period which has elapsed since April 1 last, by uniform administration under one authority and by classification, the number of inmates has been reduced, and whereas the number of inmates on each Friday night during 1910 and 1911 was 1,099 and 992 respectively, the average number during the period from April 1 to December 31 last was only 594. This reduction in the number of inmates has enabled the Board to close seven wards containing accommodation for about 500 cases. So you see that if the administration of London is really taken in hand, under the existing powers which are vested in the Local Government Board, we may get at once, as we have had in the case of the casuals, a saving of something like 66 per cent., both in accommodation and expenditure, and an absolute provision by securing the co-operation, as Mr. Burns has had the wisdom to do, not only of all those connected with the casual wards, but those who are connected with the casuals and homeless persons, such as the Church Army, the Salvation Army, and the *Morning Post* Embankment Home, and several others. This has been done by the opening of an office on the Embankment, at which homeless persons who are given tickets by the police on duty apply, and from there they are passed on either to one of the casual wards of the Board, or to one of the institutions, or the voluntary agencies, as may be considered most appropriate for them. And this is the important point. The executive work in connection with this scheme is in the hands of the Asylums Board, who pay the costs, but an Advisory Committee meet at the Local Government Board, consisting of representatives of the Asylums Board, the Local Government Board, and the various voluntary agencies co-operating. So we have under this scheme gathered together all the agencies, both voluntary and Government, which exist, effecting an enormous reform and a large reduction of cost, and with an amount of comfort and security to the poor casuals which London has never known before.

### Tuberculosis in London.

The same thing has happened in reference to tuberculosis. In the matter of tuberculosis, as the papers have shown, there have been many sad cases, and necessarily there must be such cases where you have to deal with a great number of people under a new system, when nothing is ready and everything has to be prepared. The only way in which London could have adequate bed accommodation for tuberculosis was by coming to some arrangement with the Metropolitan Asylums Board. This was not, apparently, possible on the first blush, because the Act provides that, with the view to providing treatment for insured persons suffering from tuberculosis in sanatoria and other institutions with persons or local authorities other than Poor-Law authorities, having the management of sanatoria or other institutions approved by the Local Government Board, Insurance Committees might make arrangements; but they could not make arrangements with any Poor-Law authority. The Metropolitan Asylums Board was set up by the Poor Law, and therefore is a Poor-Law authority.

### A Triumph of Administrative Astuteness.

There at first was some delay, but administrative ability has overcome the delay, because Section 64 (1) of the Insurance Act provides that if a county council decide to erect sanatoria the Local Government Board may make them a grant towards the cost, and any expenses not defrayed out of the grant shall be defrayed out of the county rate. Unless the county council decide to build sanatoria they need not do so; but if they do they will be entitled to receive a grant in aid. The proviso preventing Insurance Committees making arrangements with Poor-Law authorities was inserted on the Report stage of the Bill in the Insurance Act, undoubtedly with a view to preventing insured persons being sent into Poor-Law infirmaries and such similar buildings. The special position of the Metropolitan Asylums Board as a public hospital authority for London, with some 9,000 hospital beds for fevers, etc., was decidedly overlooked, as was the important fact that these beds were provided for the public generally, and that probably only 4 per cent. of the occupants are paupers. When this fact came out, a way out of the difficulty was found by the London County Council arranging—instead of the Insurance Committee—with the Metropolitan Asylums Board; and in this way it has been possible to secure accommodation for 360 males at the Downs Sanatorium, Sutton, Surrey, and a section of the Northern Hospital, Winchmore Hill, with accommodation for 100 females and for a further 100 cases for observation and educational purposes. This makes 560 beds out of the 1,000 beds required for London, which are provided for in existing buildings. That represents a great triumph for administrative astuteness and ability, and a great saving to the London ratepayers.

### The Question of Hospital Beds for the Insured.

In London, reverting to the hospitals and applying what I have just explained to you, we have, as I said, 11,000 beds in voluntary hospitals, of which 10,000 may be treated as being available for National Insurance purposes. In the Metropolitan Asylums Board there are 20,000 beds, of which 8,000 are for adults and 4,000 for children who are sick, and 8,000 for persons who are mentally defective. The consequence is that only a small number of beds could come from the Metropolitan Asylums Board, and they, I think, will all be occupied by tuberculous cases. But we have, in addition, the Poor-Law infirmaries, which contain 17,800 beds, of which 15,000 are constantly occupied. It would be quite possible for the Local Government Board to take the same action in regard to the Poor-Law infirmaries that has been taken in regard to the casual wards; and in that way, without any great difficulty and without the expenditure to meet it of any more money on buildings, a large number of hospital beds can be made available for insured persons in what are now known as Poor-Law infirmaries, but which would then become public hospitals. That is a point which would have to be very carefully considered, and I have ventured to deal at length with the action of the Local Government Board already because it is very material, as there are already nearly 3,000 vacant beds in Poor-Law infirmaries, seeing that each infirmary is under a different authority; that the whole of them should be brought under one authority and dealt with as a whole; that their pauper element should be shed; and that we should make them into a position of preparedness to be ready to help if necessary in reference to National Insurance so far as hospital treatment is concerned.

### How to Perfect the Hospital System.

What ought the voluntary hospitals to have behind them? There is no time to be lost. We are now in the month of March, and by the end of the year the probability will be that the number of patients requiring in-patient treatment who are insured persons will be infinitely larger than the number of beds which are available in voluntary hospitals. It is stated—on what authority I do not know—that a National Insurance Amendment Act is already in preparation. If that be so, I would like to point out that to be effective that Act should contain powers in relation to hospital benefit and hospital buildings. And it should bring the position and control of Poor-Law institutions into line with the powers conferred on the Local Government Board appertaining to the Metropolis. We want powers in relation to hospital benefit, because the voluntary hospitals ought, in my judgment, while retaining absolutely their voluntary character, to be reimbursed the actual *pro rata* cost of treating as in-patients insured persons, and that will require an Act of Parliament to accomplish. We ought, further, to confer the power on the Local Government Board and municipalities throughout the country to make loans to voluntary hospital authorities for building purposes on a basis of interest at 3 per cent. per annum, *plus* 1 per cent. sinking fund, for a term of years. And in that way, without any charge to the ratepayers of this country, we could have a speedily perfected system of hospitals with the additional extensions in the way of buildings which are necessary by using the credit of the country in a legitimate way to meet the domestic needs of the people under National Insurance. The security for the loan, I ought to point out, would be the buildings *plus* a first charge on the patients' payments made by the patients resident in the buildings, for the cost of erecting which the money was advanced. If these simple adjustments, in addition to the policy which was made successful by Mr. John Burns in reference to casuals, were to be followed throughout the country, everything would be in order for a great administrator to complete the work.

### Why the Voluntary Hospitals must be Maintained.

The voluntary hospitals must be perpetuated. They are a safeguard and protection for the sick of all classes as human beings, whether in hospitals or in their own homes. English people, as free citizens, rightly expect, and invariably receive, recognition as human beings and individuals in all our hospitals. That is not the case always under a system of State hospitals. I had the opportunity last year of making a tour of a number of these institutions, and I found everywhere evidence of a tendency, often unconscious, but always present, to treat patients as material for science rather than as individuals. That is a state of affairs which the voluntary hospital protects the people of these islands from. It is their birthright to enjoy this protection, and for that reason alone, though there are many others, the voluntary system is one which no Englishman can afford to part with, but all Englishmen must combine to perpetuate, in defence of the poor and themselves.

Complete Co-operation Essential.

We want complete co-operation and the welding together of all agencies for medical relief, State and voluntary. Such co-operation can be secured by a little patience, for the time is ripe in the sense that everybody realises that the present state of affairs cannot continue unchanged. Now, it is very important that we should maintain the voluntary system of hospital support. This voluntary basis has produced a system of hospitals which, for efficiency, equipment, administration, and, on the whole, economy, is unsurpassed by any other hospital system in the world. It represents a great revenue, and the vitality of the system has never been better revealed than in the establishment of King Edward's Hospital Fund for London in 1897, and the League of Mercy two years later. But the time has come, having regard to the abuses and the evils, and the existing want of system, when possibly there may be a measure of State aid or State grants to the voluntary hospitals for building purposes. That principle has been admitted in this country and applied to the Royal Infirmary in Manchester, where the Corporation has contributed more than once to the building fund of that institution. The Government might then gladly come forward and advance the capital sum necessary to provide pavilions for paying patients in connection with the voluntary hospitals, which are essential to the carrying out of a perfected system of medical relief for the country. Another change has recently taken place in London which shows that the trend of public opinion is all in the direction of making grants for the benefit of the population who frequent the hospitals when it can be shown to be needed for increased efficiency. The Education Authority in London has recently commenced to make grants to some medical schools attached to the voluntary hospitals in order to provide that London, as the Metropolis of the Empire, shall have a system of medical education which will be worthy of the Empire. These schools have been sadly in want of funds in recent years.

All Agencies to Co-operate together.

We need a system of co-operation which will bring into direct relation the whole of the agencies for the relief of the sick in London and throughout the country. It would speedily end hospital abuse, and would give us voluntary hospitals with classified pavilions into which all clasess of patients could be received, by payment or as free cases. Out-patient departments would consist simply of a casualty department, where every applicant could be seen on a first visit, and a Consultation Department, to which those cases could be referred who are seriously ill and require medical attention which they cannot obtain in the ordinary way from the private practitioner or dispensary. Then would the condition of the poor of this country be immeasurably improved. In a few years' time people would read with amazement of the want of organisation, of the carelessness, the overlapping, the ruinous waste of money, and the many other evils of the present system. We shall never have any changes which are adequate unless we have co-operation and reorganisation of the system.

